# Light Spectrum Differentially Affects the Yield and Phytochemical Content of Microgreen Vegetables in a Plant Factory

**DOI:** 10.3390/plants10102182

**Published:** 2021-10-14

**Authors:** Filippos Bantis

**Affiliations:** Department of Horticulture, Faculty of Agriculture, Forestry, and Natural Environment, Aristotle University, 54124 Thessaloniki, Greece; fbantis@agro.auth.gr

**Keywords:** PFAL, vertical farming, controlled environment agriculture, artificial lighting, photomorphogenesis, antioxidant content, phenolics, carotenoids, sprouts, *Brassica*

## Abstract

Light quality exerts considerable effects on crop development and phytochemical content. Moreover, crops grown as microgreens are ideal for plant factories with artificial lighting, since they contain greater amounts of bioactive compounds compared to fully-grown plants. The aim of the present study was to evaluate the effect of broad-spectra light with different red/blue ratios on the yield, morphology, and phytochemical content of seven microgreens. Mustard, radish, green basil, red amaranth, garlic chives, borage, and pea shoots were grown in a vertical farming system under three light sources emitting red/blue ratios of about 2, 5, and 9 units (RB2, RB5, and RB9, respectively). Mustard exhibited the most profound color responses. The yield was enhanced in three microgreens under RB9 and in garlic under RB2. Both the hypocotyl length and the leaf and cotyledon area were significantly enhanced by increasing the red light in three microgreens each. Total soluble solids (Brix) were reduced in 4 microgreens under RB2. The total phenolic content and antioxidant capacity were reduced under RB2 in 6 and 5 microgreens, respectively. The chlorophylls were variably affected but total the carotenoid content was reduced in RB9 in three microgreens. Overall, light wavelength differentially affected the microgreens’ quality, while small interplays in spectral bands enhanced their phytochemical content.

## 1. Introduction

A plant factory with artificial lighting (PFAL) is an innovative system for food production that utilizes modern technological advancements. Plant factories have received a high level of attention from stakeholders and researchers. During crop production in PFALs, environmental conditions can efficiently be controlled, and thus the system is ideal for off-season production [1]. Moreover, PFALs can utilize renewable energy systems and reduce their environmental impact, while vertical farming can also be accomplished, leading to considerable land-use efficiency. The latter allows PFAL construction in unused establishments within city limits, in locations that are difficult to cultivate, or in remote regions [2].

Besides, artificial lighting (as stated in their name) is an essential component of PFAL systems. Light plays two important roles in plant development: it provides the necessary energy for the photosynthetic processes, while it also acts as a signal for photomorphogenesis (i.e., plant responses to light). Light quality in particular influences several plant responses and triggers developmental and physiological modifications [3]. Plants have developed pigments and photoreceptors that distinguish the light spectra and participate in the signaling processes [4]. Pigments such as chlorophylls (*a* and *b*), which capture energy and mainly absorb red and blue lights, and carotenoids, which act as accessory molecules and contribute to photosynthetic energy transduction, are essential for photosynthesis and other physiological processes [5]. Photoreceptors from different families exhibit independent activities but they also show synergistic effects [6].

Crops for vertical farming applications must not exceed 30 cm in height in order to enable multi-layer placement; they should grow in high density, and offer added value [1]. Plants cultivated as microgreens are ideal for such applications, since they grow for a short period of time, while also containing higher amounts of phytochemical compounds compared to their respective fully-grown plants [7]. Product quality, including antioxidant content, can be manipulated by controlling light parameters, such as quantity, spectrum, and duration [8]. For example, basil seedlings accumulated more phenolics under increasing blue light composition [9]. In lettuce, carotenoids such as beta-carotene, lutein, neoxanthin, violaxanthin, and zeaxanthin were increased under supplementary blue light [10]. Moreover, *Brassica* microgreens (kohlrabi, mustard, mizuna) accumulated greater carotenoid amounts under lower light intensities [11].

To this end, the aim of the present study was to evaluate the effect of broad-spectra light sources emitting different red/blue ratios on the yield, morphology, and phytochemical content of seven popular microgreens grown in a plant factory under controlled conditions. Specifically, mustard (*Brassica juncea*), radish (*Raphanus raphanistrum*), green basil (*Ocimum basilicum*), red amaranth (*Amaranthus tricolor*), garlic chives (*Alium schoenoprasum*), borage (*Borago officinalis*), and pea shoots (*Pisum sativum*) were selected due to their nutritional value [12] as well as their heterogeneity in color, and morphological characteristics, factors that were expected to be differentially affected by light quality treatments [13].

## 2. Results

### 2.1. Morphology

**Figure 1:**
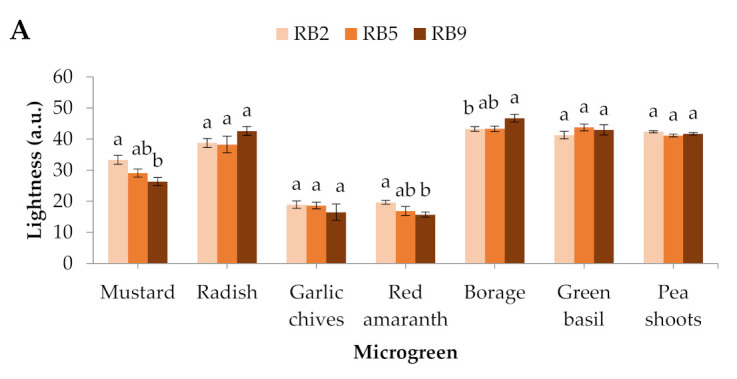

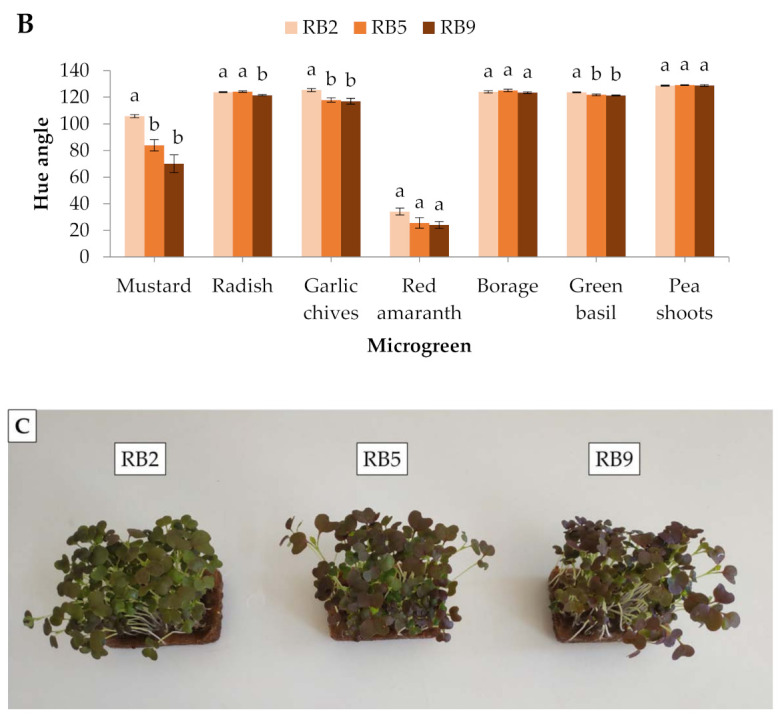
Colorimetric parameters (**A**) lightness and (**B**) hue angle of seven microgreens grown in a plant factory under three light treatments. (**C**) Mustard microgreens showing the most profound responses to light spectra among the studied species. Within each microgreen species, bars (± SE) followed by different letters are significantly different (*p* ≤ 0.05). Mean values were computed from *n* = 24 measurements. RB2: red/blue ratio = 2; RB5: red/blue ratio = 5; RB9: red/blue ratio = 9; a.u.–arbitrary unit.

In mustard and red amaranth, lightness was higher in RB2 (red/blue ratio = 2) than RB9 (red/blue ratio = 9), whereas in borage it was higher in RB9 than in RB2. No significant differences were detected in the rest of the microgreens (Figure 1A). The hue angle showed significant differences in 4 out of 7 microgreens, except for red amaranth, borage, and pea shoots. In mustard, garlic chives, and green basil, the hue angle was greater in RB2-treated microgreens compared to RB5 (red/blue ratio = 5) and RB9, while in radish it was the lowest under RB9 compared to RB2 and RB5 (Figure 1B). In general, mustard showed the most profound responses to light spectra among the studied species (Figure 1C).

**Figure 2:**
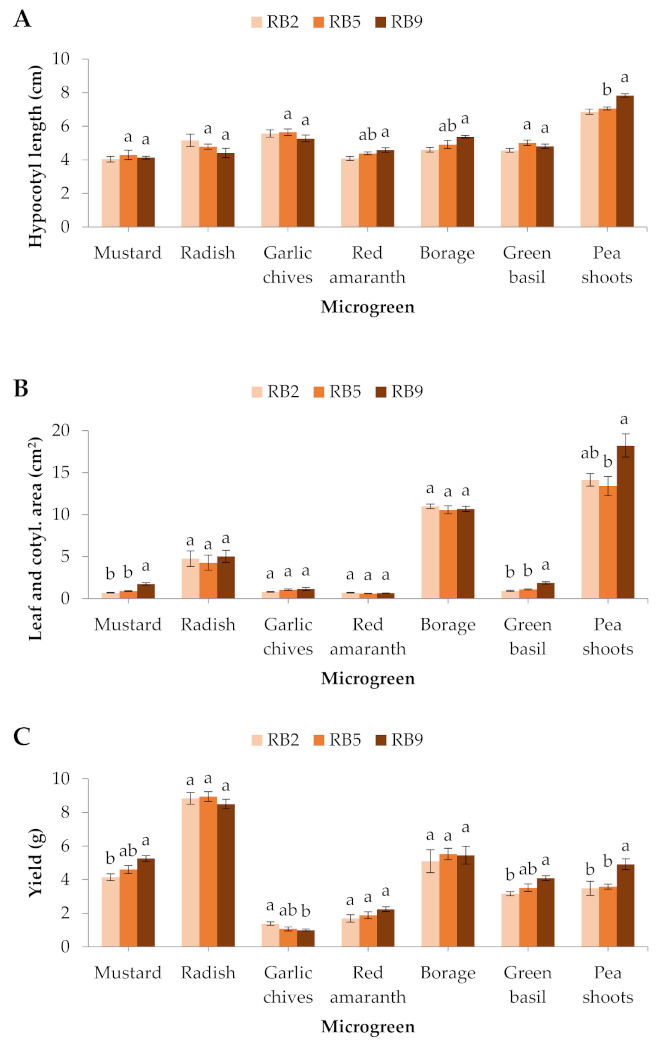
(**A**) Hypocotyl length, (**B**) leaf and cotyledon area, and (**C**) yield of seven microgreens grown in a plant factory under three light treatments. Within each microgreen species, bars (± SE) followed by different letters are significantly different (*p* ≤ 0.05). Mean values were computed from *n* = 24 (hypocotyl length, and leaf and cotyledon area) or *n* = 6 (yield) measurements. RB2: red/blue ratio = 2; RB5: red/blue ratio = 5; RB9: red/blue ratio = 9.

Red amaranth and borage formed significantly longer hypocotyls in RB9 compared to RB2, while hypocotyl length in pea shoots was significantly greater in RB9 than RB2 and RB5. No significant differences were exhibited in the rest of the microgreens (Figure 2A).

The different light treatments did not significantly affect the leaf and cotyledon area of the radish, the garlic chives, the red amaranth, or the borage. However, the mustard and green basil developed significantly larger cotyledons in RB9 compared to RB2 and RB5, while the pea shoots treated with RB9 showed significantly larger leaves than the RB5-treated microgreens (Figure 2B).

In the mustard and green basil, the total yield was significantly greater under RB9 compared to RB2, while in the pea shoots it was also greater under RB9 compared to RB2 and RB5. On the contrary, the garlic chives produced significantly greater fresh weight under RB2 than RB9. The rest of the microgreens were not affected (Figure 2C).

### 2.2. Total Soluble Solids

**Figure 3:**
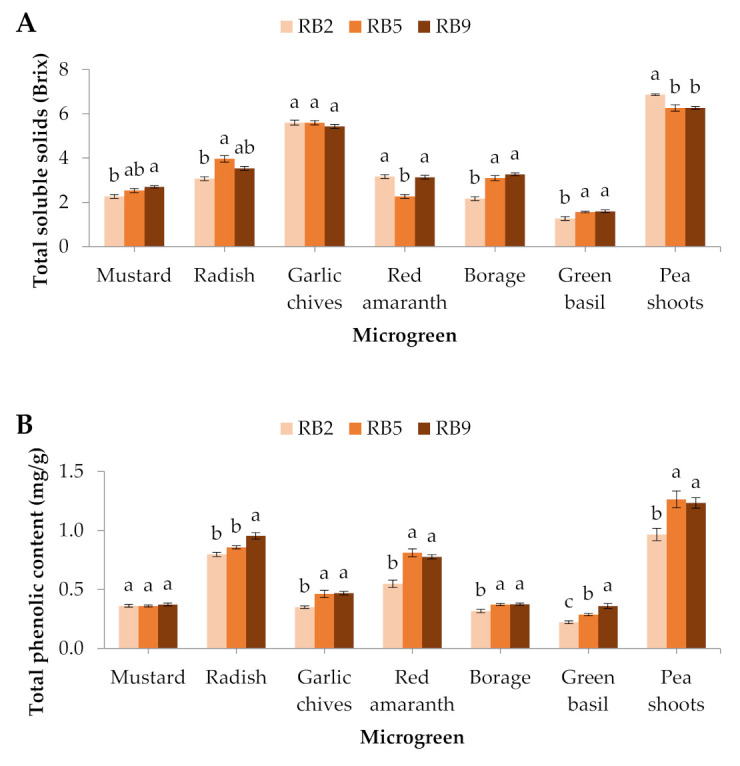

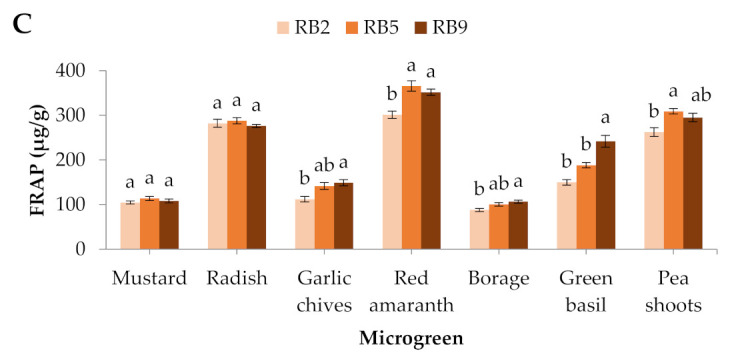
(**A**) Total soluble solids, (**B**) total phenolic content, and (**C**) antioxidant capacity (FRAP) of seven microgreens grown in a plant factory under three light treatments. Within each microgreen species, bars (±SE) followed by different letters are significantly different (*p* ≤ 0.05). Mean values were computed from *n* = 3 measurements. RB2: red/blue ratio = 2; RB5: red/blue ratio = 5; RB9: red/blue ratio = 9.

The total number of soluble solids showed significant differences in all the microgreens except for the garlic chives.Specifically, in the mustard, radish, borage and green basil, the total of soluble solids was the lowest under RB2, in red amaranth it was the lowest under RB5, whereas in pea shoots it was the greatest under RB2 (Figure 3A).

### 2.3. Phytochemical Analyses

In this study, the total phenolic content was significantly affected in 6 out of 7 microgreens, except for mustard. In the garlic chives, red amaranth, borage, green basil, and pea shoots, the total phenolic content was the lowest in RB2-treated microgreens compared to the other light treatments, while in radish, RB9 induced the greatest total phenolic content compared to RB2 and RB5 (Figure 3B).

Quite similarly to the phenolic compounds, the antioxidant potential displayed by ferric reducing antioxidant power (FRAP) exhibited significantly lower values in the RB2-treated garlic chives, red amaranth, borage, and pea shoots, while green basil showed significantly higher values in RB9 compared to the other light treatments. No significant differences were observed in mustard and radish (Figure 3C).

### 2.4. Chlorophyll and Carotenoid Content

**Figure 4:**
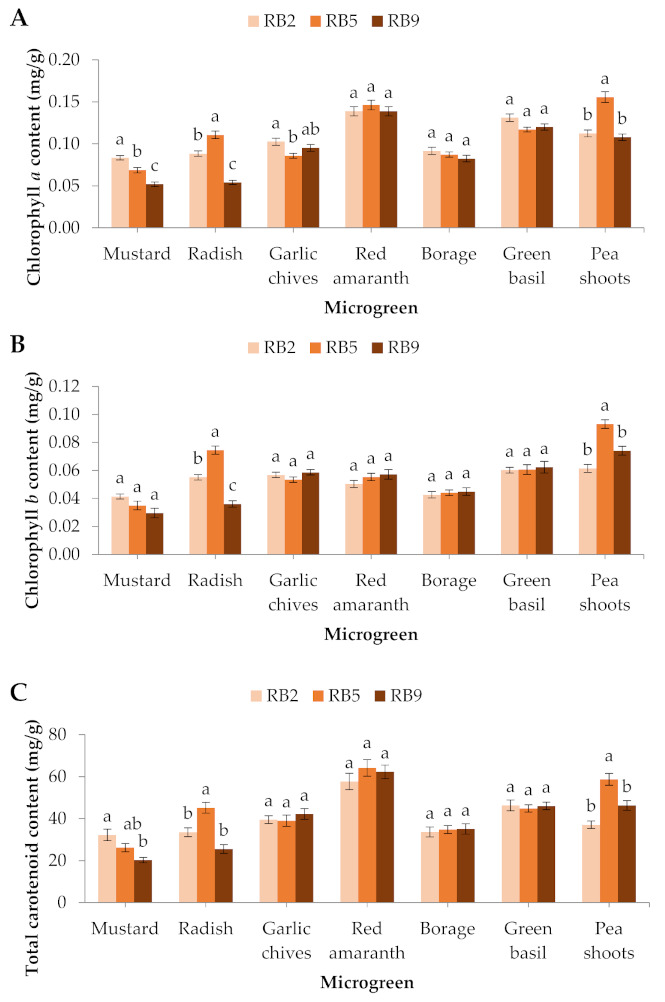
(**A**) Chlorophyll *a*, (**B**) chlorophyll *b*, and (**C**) total carotenoid contents of seven microgreens grown in a plant factory under three light treatments. Within each microgreen species, bars (± SE) followed by different letters are significantly different (*p* ≤ 0.05). Mean values were computed from *n* = 3 measurements. RB2: red/blue ratio = 2; RB5: red/blue ratio = 5; RB9: red/blue ratio = 9.

The chlorophyll *a* content in mustard was significantly greater in RB2 compared to RB5 and RB9, in the garlic chives it was significantly greater in RB2 compared to RB5, while in the radish and pea shoots chlorophyll *a* was significantly greater in RB5 compared to RB2 and RB9. No significant differences were observed in the red amaranth, the borage, or the green basil (Figure 4A). The chlorophyll *b* content was significantly affected in 2 out of 7 microgreens. Specifically, in the radish and pea shoots, the chlorophyll *b* content was significantly higher in RB5 compared to RB2 and RB9 (Figure 4B). The total chlorophyll content in the mustard was significantly greater in RB2 than RB9, while in the radish and pea shoots the total chlorophyll was significantly greater in RB5 compared to RB2 and RB9 (data not shown).

In the radish and pea shoots used in this study, the total carotenoid content was significantly greater in RB5 compared to RB2 and RB9, while in the mustard it was significantly greater in RB2 compared to RB9. No significant differences were observed in the rest of the species (Figure 4C).

## 3. Discussion

The selection of appropriate light wavelengths for certain crops is a matter that needs considerable attention from stakeholders. Currently, producers incorporate light sources emitting wavelengths that facilitate plant scouting and other practices inside the PFAL system. In the present study, RB2 (red/blue ratio = 2) and RB5 (red/blue ratio = 5) had color rendering indexes (CRIs) above 50, which enables plants’ visual examination due to its white or whitish perception by the human eye. Moreover, growth and development are affected in a variable manner by light quality, depending on the plant’s genotype [14]. Indeed, the quality and phytochemical characteristics of seven microgreens were differentially affected by light sources that emitted different red/blue ratios.

### 3.1. Morphology

Color is an essential factor in vegetable selection by consumers. Leaves and cotyledons with deep green or red colors are usually regarded as highly nutritious and has having increased antioxidant capacity [15,16]. In the present study, mustard in particular had the most profound color differences, with reddish cotyledons under RB9 and green cotyledons under RB2, which were easily detectable by sight. This observation is essential for the production of a popular commodity with intense color and for greater acceptance by consumers. Similarly, Kong and Zheng [17] found a greater hue angle in mustard (*Brassica juncea*) cotyledons grown under monochromatic blue and blue-containing LEDs than under monochromatic red light.

Hypocotyls are major qualitative components of microgreens as well as a large portion of their edible parts. Long cotyledons are often perceived as indexes of high quality; thus, they are more attractive for producers and consumers [16]. RB9 enhanced the hypocotyl length in 3 microgreens in this study. In a study with *Brassica* microgreens, the authors found shorter hypocotyls in mustard (*Brassica juncea*) and kale (*Brassica oleracea*), with decreasing red/blue ratios; and even in the same family, species dependency was evident in growth parameters such as leaf area and fresh mass [18]. Moreover, three lettuce (*Lactuca sativa*) cultivars (Mantecosa, Angel, and Romana) showed greater height under treatment with increased red light [19]. Hypocotyl elongation and cotyledon expansion are typical shade-avoidance responses triggered by phytochrome photoreceptors under certain red/far-red ratios [20]. In particular, phytochrome exists in two interconvertible forms, Pr and Pfr, depending on the ratio of red and far-red wavelengths on the light spectrum. Increased far-red on the light spectrum typically leads to a number of shade-avoidance responses, such as those mentioned above. Specifically, phytochromes, along with cryptochromes (i.e., blue- and UV-absorbing photoreceptors), have been found to regulate the transcription factors HYH and HY5, which induce photomorphogenesis, and COP1, which suppresses photomorphogenesis [18,21,22]. In the present study, RB2 had a higher red/far-red ratio compared to RB9 (red/blue ratio = 9), which possibly explains the lower hypocotyl length and the leaf and cotyledon area under the former light treatment [23]. Even though the three light treatments displayed similar phytochrome photostationary states (PPSs), the responses related to phytochrome activity were considerable in three of the tested microgreens. Apart from red and far-red wavelengths, blue light also affects extension growth. Specifically, blue light acting through CRY1 (a cryptochrome photoreceptor) is known to repress hypocotyl elongation via gibberellin and auxin regulation [24]. RB2 emits the highest amount of blue wavelength and is possibly responsible for the lowest hypocotyl length in three microgreens.

The area of cotyledons and leaves, as well as chives in the case of garlic, is the second component of the microgreens’ edible parts. Along with hypocotyl, the area is an essential parameter defining its market selection and acceptance, since expanded cotyledons and leaves are associated with greater yields and better overall quality. Both parameters can efficiently be controlled by manipulating light quality, especially red and blue wavelengths [25]. As with the hypocotyl length, the area of leaves and cotyledons was promoted by RB9 in three microgreens. In a study with arugula, cabbage, kale, and mustard, all the microgreens developed larger cotyledons when treated with monochromatic red light compared to blue-containing LEDs such as monochromatic blue, blue-green, and blue-UV [17]. Mishra and Khurana [26] stated that each plant species requires a minimum threshold of blue light to trigger shade-avoidance responses. This statement might explain the variation in microgreen responses to the same light wavelength.

According to McCree [27], red and blue are the most efficient wavelengths for driving photosynthesis and contribute to CO_2_ fixation and biomass production. These wavelengths are absorbed by pigments of the primary (i.e., chlorophylls) or secondary (i.e., anthocyanins and carotenoids) metabolism. As was the case in this study, other research findings are contradictory regarding the fresh biomass production of various plants. For example, sweet basil (*Ocimum basilicum*) developed more fresh weight under a red/blue ratio of 2 or 3 units compared to 0.5, 1, and 4 units [28]. Mustard and kale microgreens reportedly had greater fresh weight under monochromatic blue compared to red/blue ratio of 1 [17], while the opposite result was observed in *Brassica carinata* microgreens [29]. Moreover, Gerovac et al. [30] found greater fresh weight in mustard microgreens grown under red/green/blue (74/18/8%) compared to red/blue (87/13%) and red/blue/far-red (84/9/7%), while they did not find significant differences in the fresh weight of kohlrabi and mizuna microgreens. Light-absorbing film with peak emission at 600 or 660 nm induced 11% greater edible fresh mass in lettuce compared to no film [31]. In another study, the treatment of tomato transplants with supplementary light at 595 nm led to a decreased leaf area, and shoot and root dry weight compared to basal light [32].

### 3.2. Total Soluble Solids

Qualitative parameters, including phytochemical compounds, directly affect the economic value of vegetables [33]. By determining the total soluble solids of microgreens, it is possible to quantify the tastiness of these commodities, an important aspect for consumers. In a study with spinach (*Spinacia oleracea*), baby leaves grown under broad-spectrum LEDs did not exhibit significant differences in soluble sugar content [34]. However, lettuce (*Lactuca sativa*) treated with white-red light produced greater amounts of sugars compared to white-blue light [35]. In addition, a study with lamb’s lettuce (*Valerianella locusta*) revealed greater sugar content under a 90% red-10% blue light compared to monochromatic red, monochromatic blue, and red-blue treatments [36], proving the necessity of red light in relatively high portions for the accumulation of sugars.

### 3.3. Phytochemical Analyses

Plants produce antioxidant compounds, such as phenolics, in response to environmental stress factors as a means of adaptation in various biotic and abiotic constraints that would otherwise damage the photosynthetic apparatus [37]. Light quality is among the environmental parameters that trigger the biosynthesis of these compounds [38]. In the present study, it was clear that increasing blue light portion induced the accumulation of decreased phenolic compounds in six microgreens. Blue, red, and far-red wavelengths have the ability to regulate the biosynthesis of phenolic compounds in a direct or indirect manner through signaling, which leads to the expression of key enzymes, or through increasing shikimic acid, a precursor of phenolic compounds [39,40]. Moreover, the activity of a major enzyme participating in the phenolic biosynthesis, phenylalanine ammonia lyase (PAL), is known to be regulated by light quality [41]. *Brassica carinata* microgreens produced greater amounts of phenolics when treated with monochromatic blue compared to red and 50% red/ 50% blue light [29]. As with the green basil used in this study, Lobiuc et al. [42] found a higher phenolic content in green basil (*Ocimum basilicum*) under increased red light portion, while Gimenez et al. [40] reported greater total phenolic content in purslane microgreens treated with red-blue light compared to fluorescence and red-blue, including far-red. Romaine baby leaf lettuce had significantly higher phenol, but lower anthocyanin, and tocopherol amounts under supplemental light with a peak at 622 nm, and less ascorbic acid and tocopherols under supplemental light, with a peak at 595 nm [43].

Regarding the antioxidant capacity, five out of seven microgreens exhibited lower values with increasing blue portions, as observed with phenolic compounds. On the contrary, Pennisi et al. [30] reported significantly greater antioxidant capacity (ferric reducing antioxidant power: FRAP) in basil plants treated with a red/blue ratio of 2 units compared to those treated with 0.5 or 1, while total flavonoid concentration was promoted under a red/blue ratio of 3 units compared to ratios ranging from 0.5 to 4 units. In a study with purslane (*Portulaca oleracea*) microgreens, antioxidant capacity was not affected by fluorescent, red-blue, or red-blue, including far-red lights [40].

### 3.4. Chlorophyll and Carotenoid Content

Chlorophylls are the major pigments responsible for photosynthesis and subsequent carbon fixation. Both chlorophylls *a* and *b* absorb wavelengths in the red and blue parts of the visible spectrum [5]. In this study, chlorophylls *a* and *b* were reduced under RB9 in three and two microgreens, respectively. The main photoreceptor involved in chlorophyll biosynthesis, cryptochrome, mainly absorbs blue light and its action is connected with red- and far-red-absorbing phytochrome photoreceptors [6]. Stutte et al. [44] reported that PPS values above 0.6 promoted the phytochrome response in most plants. In this study, all the light treatments had PPS values of 0.82 and 0.83. In addition, blue light induces the expression of genes that participate in chlorophyll formation, such as *MgCH*, *GluTR*, and *FeCH* [45], and controls a number of enzymes participating in chlorophyll biosynthesis, such as aminolevulinic acid (ALA)-synthase, ALA-dehydratase, dioxovalerate (DOVA)-dehydrogenase, and DOVA-transaminase [42,46].By contrast, red light is responsible for the reduction of 5-aminolevulinic acid, a necessary precursor of chlorophyll biosynthesis [45]. In the present study, the light treatment emitting the lowest amount of blue light (i.e., RB9) induced the production of the fewest chlorophylls in three microgreens, while two more showed a tendency for reduced (non-significant) values under the same treatment. The chlorophyll index of basil (*Ocimum basilicum*) “Genovese” was greater when plants were treated with a red/blue ratio of 2 or 3 units compared to 0.5, 1, and 4 units [28]. Toscano et al. [47] found that red amaranth grown under monochromatic blue produced greater amounts of chlorophyll *a*, total chlorophyll, and carotenoids compared to white (21% blue; 38% green; 35% red; 6% far-red) and monochromatic red, while turnip greens did not show a response. In addition, the percentage of blue light did not affect chlorophyll *a*, *b*, or the total chlorophyll of four *Brassica* microgreens, including mustard, while the authors observed a species-dependent effect during the first stage of plant growth [48].

Carotenoids are accessory pigments responsible for excess energy dissipation and are related to the antioxidant mechanism [49]. Three microgreens had a lower carotenoid content under the effect of RB9. According to Planck’s law (E = h × c × λ − 1), within the visible spectrum, blue light has a shorter wavelength, and thus higher energy, compared to green and red wavelengths. Moreover, CRY2 protein has been shown to participate in carotenoid biosynthesis through a blue-light dependent mechanism [50]. In three microgreens, RB2 (17.50% blue) and RB5 (11.38% blue) enhanced carotenoid accumulation compared to RB9 (7.62% blue), which mostly emits red and far-red light. However, the relatively low but highly efficient photosynthetic photon flux density (PPFD: 180 ± 10 μmol m^−2^ s^−1^) probably did not have a damaging effect on most of the microgreen species, so did not alter their carotenoid content. In two studies, *Brassica* microgreens and basil showed no significant response under the influence of different red/blue ratios [42,48]. Similarly to the radish and pea shoots in our study, three cultivars of microgreen and baby leaf lettuce treated with a wavelength comparable to RB5 produced greater amounts of β-carotene, lutein, and total carotenoids compared to treatments with higher blue portions [19]. Using a non-destructive method for carotenoid determination, Brazaityte et al. [18] found a lower carotenoid reflectance index in kale and mustard treated with increased blue light. Moreover, Samuoliene et al. [50] reported that 16% blue light is the optimum portion for enhanced lutein, neoxanthin, and violaxanthin biosynthesis in mustard, beet, and parsley. As suggested by various researchers (e.g., [11,50]), carotenoid production and accumulation is a species-dependent response that is variably affected by the plant genotype.

## 4. Materials and Methods

### 4.1. Plant Material and Sowing

The experiment was conducted in a PFAL facility located in Thessaloniki, Greece. Seeds from seven plant species, popularly grown as microgreens, were bought from CN Seeds (Pymoor, Ely, Cambridgeshire, United Kingdom). Specifically, the species examined were mustard (*Brassica juncea* cv. Red Lion), radish (*Raphanus raphanistrum* cv. Saxa), green basil (*Ocimum basilicum* cv. Sweet Genovese), red amaranth (*Amaranthus tricolor* cv. Red Aztec), garlic (*Alium schoenoprasum* cv. Thick Leaf), borage (*Borago officinalis* cv. Blue), and pea (*Pisum sativum* cv. Dun).

The seeds were sown and watered in plastic pots (5 × 8 cm) filled with cannabis mat, a hydroponic substrate that allows the seeds to sit on top and grow quickly without decelerating their ascent towards the light, which is crucial for the short-timed microgreen production. Prior to sowing, the pea shoots were soaked in water for 24 h. Six pots per species and light treatment were placed in a larger container (17 × 33 cm) that was able to fit 12 pots. In total, 126 pots (6 pots × 7 species × 3 light treatments) were distributed among 12 containers. The species were placed in the larger containers in pairs depending on their growing speed and expected day of harvest. Specifically, the pairs were mustard-radish, green basil-red amaranth, and garlic chive-borage, while the pots of pea shoots were placed in an individual container due to their faster growth. Regarding nutrient provision, Hoagland solution (pH 6.5; electric conductivity 2.6 mS cm^−1^) [51] was added to the containers. It was absorbed by the plants’ capillaries through holes in the pots. Subsequently, a lid was placed on top of the containers and the plants remained in darkness until germination. Table 1 displays the sowing density as well as important days, such as the end of dark period (beginning of lighting phase) and the day of harvest for each microgreen.

### 4.2. Growth and Light Conditions

**Figure 5:**
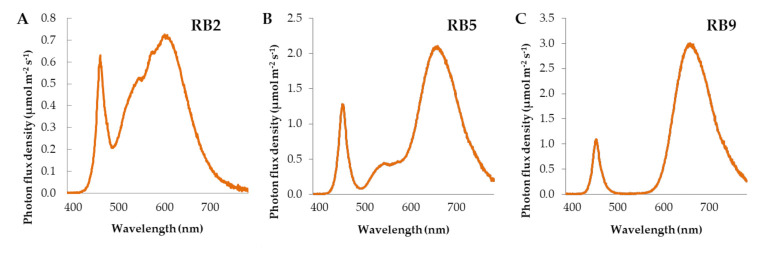
Spectral distribution of (**A**) RB2, (**B**) RB5, and (**C**) RB9 LED light treatments. RB2: red/blue ratio = 2; RB5: red/blue ratio = 5; RB9: red/blue ratio = 9.

Upon germination and after the microgreens reached 3 cm in height, the lids were removed and the containers were placed on the shelves (0.4 × 1.2 m) of a three-shelf rack. Each shelf was illuminated by a different light-emitting diode (LED) fixture (120 cm, 132 W), providing a broad-spectra wavelength. Briefly, RB2 (red/blue ratio = 2) emitted the highest amount of blue and green lights, the blue and red lights peaked at 454 and 600 nm, respectively, while the fixture had the highest color rendering index (CRI). RB5 (red/blue ratio = 5) emitted intermediate amounts of blue, green, red, and far-red wavelengths compared to the other treatments. RB9 (red/blue ratio = 9) emitted the highest amount of red and far-red lights and, along with RB5, the blue and red lights peaked at 448 and 660 nm, respectively. The RB2 was manufactured by V-TAC EUROPE (Sofia, Bulgaria). The RB5 and RB9 were manufactured by Valoya OY (Helsinky, Finland). The wavelength distribution and important light parameters were measured with HD 30.1 spectroradiometer (DeltaOhm Srl, Padova, Italy) and are displayed in Table 2 and Figure 5. The photoperiod was 16 h day/8 h night and the photosynthetic photon flux density was 180 ± 10 μmol m^−2^ s^−1^. Temperature was 22 ± 1 °C, the relative humidity was 65 ± 10%, and air recirculated through two fans placed on the roof and the ground, respectively, while the room was also ventilated.

### 4.3. Measurements and Analyses

The whole plants were cut 1 cm above the substrate, and measurements and analyses followed. For the total number of soluble solids, the phytochemical analysis, and the chlorophyll and carotenoid content determinations, each sample consisted of all the plants (see Table 1) from two randomly selected pots per light treatment, leading to a total of three samples.

#### 4.3.1. Morphology

Upon harvesting, microgreens were weighted in order to obtain their yield (i.e., six samples consisting of all the plants from each pot per light treatment). The hypocotyl length was measured in 24 plants per species and light treatment (i.e., 4 plants from each pot) using a Vernier caliper, and the leaf and cotyledon area was measured using a leaf area meter (LI-3000C, LI-COR biosciences, Lincoln, NE, USA). Specifically, the cotyledons or leaves from 24 plants per species and light treatment (i.e., 4 plants from each pot) were removed and meticulously laid onto a transparent surface, which was dragged across the leaf area meter’s measuring unit. In addition, the colorimetric parameters (i.e., the lightness and hue angle) were determined using a CR-400 Chroma Meter (Konica Minolta Inc., Tokyo, Japan).

#### 4.3.2. Total Soluble Solids (Brix)

The microgreens were stored at −30 °C for a few days until the phytochemical analyses. The plants were then homogenized and the total number of soluble solids (expressed as Brix) was immediately measured with a refractometer (PAL-α, Atago, Tokyo, Japan). Brix can be associated with sugar content in leafy vegetables, as has been observed for cabbage (*Brassica oleracea*) [53].

#### 4.3.3. Phytochemical Analysis

The method of Singleton and Rossi [54] was used to determine the total phenolic content. The phenolic compounds were extracted with 80% aqueous methanol. Aliquot 0.5 mL extract, 2.5 mL of 10% Folin–Ciocalteau’s reagent, and 2 mL of 7.5% Na_2_CO_3_ were incubated in a water bath (5 min at 50 °C), and the absorbance was measured at 760 nm using a spectrophotometer. The total antioxidants were determined using a ferric-reducing antioxidant power (FRAP) assay. An aliquot 0.1 μL extract and a 3 mL working solution (CH_3_COONa pH 3.6, TPTZ, and FeCl_3_) were incubated in a water bath (4 min at 37 °C), and the absorbance was immediately measured at 593 nm [55].

#### 4.3.4. Chlorophyll and Carotenoid Content

Chlorophylls and carotenoids were extracted with 80% aqueous acetone. After two centrifuges (4 °C, 10.000 rpm, 10 min), the absorbance was measured at 470, 647, and 663 nm [56]. The chlorophyll *a* and *b* and carotenoid concentrations were calculated as follows:

Chl *a* = 12.25 × A_663.2_ – 279 × A_646.8_ (1)

Chl *b* = 21.50 × A_646.8_ – 5.10 × A_663.2_ (2)

Carotenoids = (1000 × A_470_ − 1.82 × Chl *a* − 85.02 × Chl *b*)/198 (3)

Abbreviated terms in Equations (1)–(3): Chl *a*: chlorophyll *a*; Chl *b*: chlorophyll *b*; A_663.2_: absorbance at 663.2 nm; A_646.8_: absorbance at 646.8 nm; A_470_: absorbance at 470 nm.

#### 4.3.5. Experimental Design and Statistical Analysis

Each light treatment was allocated a shelf on a vertical rack. Six plastic pots per species were placed in a larger container, which provided water and nutrients through holes in the bottom. The containers were placed in the same position on each shelf; thus, each species was placed at the same vertical level on the three shelves. For the yield determination, each pot was considered a repetition (six repetitions in total). For the hypocotyl length and leaf area determinations, four microgreens per pot (24 repetitions in total) were used. Due to the low fresh mass in a single pot, two pots per repetition (three repetitions in total) were used for the total number of soluble solids, the phytochemical analyses, and the chlorophyll and carotenoid content determinations.

The experiment was performed twice and similar conclusions were drawn in both; thus, the results from the first repetition are presented. The analysis of variances was conducted using an IBM SPSS statistical package (SPSS 25.0, IBM Corp., Armonk, NY, USA). The post-hoc analysis was conducted with the method of Tukey at significance level α = 0.05.

## 5. Conclusions

The tested light sources emitted broad light with varying red/blue ratios and affected seven microgreens in a variable manner. A major visual characteristic, leaf color portrayed by lightness and hue angle, was mainly affected in mustard, where reddish leaves under RB9 (red/blue ratio = 9) were observable even by the naked eye. The hypocotyl length, the leaf and cotyledon area, and the yield were enhanced by increasing the red light portion in three microgreens. As a general rule, increasing the blue light portion led to lower phenolics and total antioxidant compounds, as displayed in six and five microgreens respectively. By contrast, the chlorophyll and carotenoid contents decreased under RB9. The red peak wavelength of RB2 (red/blue ratio = 2) was 600 nm compared to those of RB5 (red/blue ratio = 5) and RB9 with 660 nm. Research is scarce regarding the effects of these wavelengths on plant development and quality. Moreover, it is possible that the short irradiation time (5–8 days depending on the microgreen species) was insufficient for the different light qualities to have a significant effect on some of the characteristics of certain microgreens, even though the red and blue portions of each light treatment were far apart.

## Figures and Tables

**Table 1:** Sowing density, end of dark period, and day of harvest of seven microgreens grown in a plant factory; d–days.

Microgreen	Sowing Density *	End of Dark Period (d)	Harvest (d)
Mustard	300	3	8
Radish	250	3	8
Green basil	600	4	12
Red amaranth	1500	4	12
Garlic chives	500	4	12
Borage	80	4	12
Pea shoots	12	3	10

* Sowing density refers to the approximate number of seeds sowed in a 5 × 8 cm plastic pot.

**Table 2:** Spectral distribution (percentages of total photons), red/blue ratio, red/far-red ratio, photosynthetic photon flux density (PPFD), yield photon flux density (YPFD), correlated color temperature (CCT), color rendering index (CRI), and phytochrome photostationary state (PPS) for the light treatments tested. RB2: red/blue ratio = 2; RB5: red/blue ratio = 5; RB9: red/blue ratio = 9. PPS and YPFD were calculated according to Sager et al. [52].

Parameters	Light Treatment		
	RB2	RB5	RB9
UV %; 380–399 nm	0.03	0.02	0.02
Blue %; 400–499 nm	17.50	11.38	7.62
Green %; 500–599 nm	43.84	13.85	2.34
Red %; 600–699 nm	35.40	56.48	67.25
Far-red %; 700–780 nm	3.23	18.28	22.77
Blue peak (nm)	454	448	448
Red peak (nm)	600	660	660
Red/Blue ratio	2.02	4.97	8.82
Red/Far-red ratio	6.44	3.09	2.95
PPFD (μmol m^−2^ s^−1^)	180 ± 10	180 ± 10	180 ± 10
YPFD (μmol m^−2^ s^−1^)	84.8	75.4	73.8
CCT (K)	4105	2143	-
CRI	85.5	71.0	-
PPS	0.83	0.82	0.82

## Data Availability

Not applicable.
